# Brain functional connectivity‐based prediction of vagus nerve stimulation efficacy in pediatric pharmacoresistant epilepsy

**DOI:** 10.1111/cns.14257

**Published:** 2023-05-11

**Authors:** Hao Chen, Yi Wang, Taoyun Ji, Yuwu Jiang, Xiao‐Hua Zhou

**Affiliations:** ^1^ Beijing International Center for Mathematical Research Peking University Beijing China; ^2^ Department of Pediatrics and Pediatric Epilepsy Center Peking University First Hospital Beijing China; ^3^ Department of Biostatistics, School of Public Health Peking University Beijing China; ^4^ Pazhou Lab Guangzhou China

**Keywords:** brain functional connectivity, ictal EEG recording, scalp EEG, support vector machine, vagus nerve stimulation

## Abstract

**Objective:**

Although vagus nerve stimulation (VNS) is a common and widely used therapy for pharmacoresistant epilepsy, the reported efficacy of VNS in pediatric patients varies, so it is unclear which children will respond to VNS therapy. This study aimed to identify functional brain network features associated with VNS action to distinguish VNS responders from nonresponders using scalp electroencephalogram (EEG) data.

**Methods:**

Twenty‐three children were included in this study, 16 in the discovery cohort and 7 in the test cohort. Using partial correlation value as a measure of whole‐brain functional connectivity, we identified the differential edges between responders and nonresponders. Results derived from this were used as input to generate a support vector machine‐learning classifier to predict VNS outcomes.

**Results:**

The postcentral gyrus in the left and right parietal lobe regions was identified as the most significant differential brain region between VNS responders and nonresponders (*p* < 0.001). The resultant classifier demonstrated a mean AUC value of 0.88, a mean sensitivity rate of 91.4%, and a mean specificity rate of 84.3% on fivefold cross‐validation in the discovery cohort. In the testing cohort, our study demonstrated an AUC value of 0.91, a sensitivity rate of 86.6%, and a specificity rate of 79.3%. Furthermore, for prediction accuracy, our model can achieve 81.4% accuracy at the epoch level and 100% accuracy at the patient level.

**Significance:**

This study provides the first treatment response prediction model for VNS using scalp EEG data with ictal recordings and offers new insights into its mechanism of action. Our results suggest that brain functional connectivity features can help predict therapeutic response to VNS therapy. With further validation, our model could facilitate the selection of targeted pediatric patients and help avoid risky and costly procedures for patients who are unlikely to benefit from VNS therapy.

## Graphical abstract

This study provides a treatment response predictive model for vagus nerve stimulation (VNS) using scalp electroencephalogram (EEG) data with seizure recordings and offers new insights into its mechanism of action. Our results suggest that brain functional connectivity features derived from ictal states of scalp EEG data can help predict therapeutic response to VNS therapy.

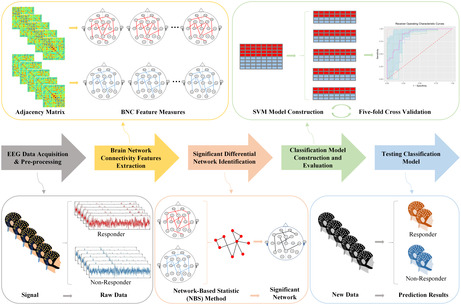

## 1 INTRODUCTION

Epilepsy is one of the most common chronic neurological disorders affecting people of all ages worldwide.^1^ According to the World Health Organization (WHO), approximately 70 million people suffer from epilepsy,^2^ and the annual cumulative incidence rate of epilepsy is over 700 per 100,000 individuals,^3^ which is higher in children than in young and middle‐aged adults.^4^ The brain of a child is highly plastic and still developing. Recurrent episodes of epileptic brain activity can permanently change the brain's shape and alter neural circuits, leading to lifelong mental, psychological, and motor problems.^5^ Therefore, effective treatment of pediatric epilepsy is essential in that it may improve pediatric patients' subsequent quality of life. However, more than one third of children with epilepsy do not respond to any antiseizure medications (ASMs).^6^, ^7^ These children are considered to have pharmacoresistant epilepsy and adjunctive therapies, including vagus nerve stimulation (VNS), might be better treatment strategies for them.

Vagus nerve stimulation – a low‐risk neuromodulation therapy for children with drug‐resistant epilepsy – attempts to control seizures by sending small electrical pulses from the vagus nerve to the brain. Many studies have demonstrated that VNS therapy is effective and safe in reducing seizures in adults and children.^8^, ^9^, ^10^ Despite the widespread availability of VNS, its reported efficacy varies in pediatric patients,^11^, ^12^ and there is still uncertainty in predicting those that will respond to it. Identifying responses to VNS in pediatric patients is critical to prevent surgical risks, save time and money for nonresponders, and help responders receive this effective VNS therapy in time.

As epilepsy has been considered a network disorder^13^ and VNS prevents or lessens seizures by sending pulses of electrical energy to different brain regions via the vagus nerve, brain network‐based biomarkers appear to be the most promising tool for identifying VNS responders. In addition, the rationality of such biomarkers has been confirmed by many previous studies.^14^, ^15^, ^16^, ^17^, ^18^, ^19^, ^20^, ^21^ Most such studies investigated differences in measures of functional brain connectivity in children undergoing VNS treatment and showed that brain network characteristics were often altered in children with intractable epilepsy. These results suggest that the variability in VNS therapy response may be related to differences in brain connectivity between patients. However, these studies did not predict which children will benefit from VNS therapy. We herein address this deficiency.

To our knowledge, only a few studies selected brain network‐based biomarkers and used them to identify patients most likely to respond to VNS therapy. Ibrahim et al.^18^ performed the first study to utilize resting state functional MRI (rs‐fMRI) data to detect differences in brain networks between VNS responders and nonresponders, hence predicting patients that may benefit from VNS. Subsequently, Babajani‐Feremi et al.^17^ proposed predicting VNS seizure outcomes using network features derived from resting state magnetoencephalography (rs‐MEG) data. More recently, Mithani et al.^16^ developed a predictive model using combined structural and functional connectivity features derived from diffusion tensor imaging data and rs‐fMRI data to predict VNS responsive or nonresponsive children. Given this evidence, it is reasonable to use brain functional connectivity features to predict VNS seizure outcomes in patients with pediatric epilepsy.

In the current study, we developed a machine‐learning method to predict VNS seizure outcomes using brain functional connectivity features derived from scalp electroencephalogram (EEG) data acquired prior to VNS implantation. Compared to the abovementioned previous studies, ours is novel in terms of the following aspects. First, this study is the first to investigate VNS response prediction using network features derived from EEG data acquired before VNS implantation. As children tend to move while being scanned, performing fMRI or MEG scans in children can be problematic.^22^, ^23^ Therefore, EEG is a more suitable technique than MEG and fMRI to detect brain functional activity in children. EEG connectivity analysis is expected to provide a more reliable prediction of VNS response. Second, this is equally the first study to use ictal EEG recordings to identify potential VNS responders, whereas other studies have used resting state data. Scheid et al. demonstrated the plausible role of ictal EEG recordings in predicting treatment response to responsive neurostimulation therapy^24^; therefore, using ictal EEG data to predict VNS response is also expected. Third, this is the first study to employ partial correlations to calculate functional brain connectivity, which measures the direct interaction between two electrodes in the absence of a set of other electrodes. Partial correlation can prevent spurious correlations and obtain a more reliable relationship between two electrodes by controlling others compared to spatial autocorrelation used in Ibrahim et al.'s study^18^ and Pearson's correlation used in Mithani et al.'s study.^16^

## 2 METHODS

### 2.1 Participants

The patients were children who had undergone VNS implantation at the Peking University First Hospital between January 7 and December 30, 2019. Inclusion criteria were as follows: (1) diagnosis of pharmacoresistant epilepsy as defined by Kwan et al.^25^; (2) having at least six seizures per month; (3) participants' family members could understand the benefits and risks; and (4) at least one ictal EEG recordings of brain activity. The following exclusion criteria were utilized: (1) brain MRI revealed resectable lesions; (2) vagal nerve lesion or damage; (3) poor health and other contraindications to surgery; and (4) compliance deficit to record the epileptic diary, implant VNS system, and complete the postoperative 3 years of follow‐up. Eventually, 23 patients were included in the study.

The most widely used outcome of interest was the percentage reduction of seizure frequency from baseline (i.e., before VNS implantation) to their last follow‐up visit. A percentage reduction equal to 100% means that the seizures completely disappeared, while a percentage reduction <0 means that the seizure frequency increased. In most of the literature, children who exhibited a reduction in seizure frequency from a baseline of >50% were considered responders. Conversely, those with a reduction of ≤50% were considered nonresponders. Of these 23 subjects, 15 were responders and 8 were nonresponders. Ten randomly selected responders and six randomly selected nonresponders were included in the discovery cohort to train our model, while the others were included in the testing cohort.

### 2.2 EEG data acquisition and preprocessing

The EEG data used in this study were collected prior to VNS implantation and recorded using a 19‐channel EEG system according to the 10–20 system. The electrodes included FP1, FP2, F3, F4, C3, C4, P3, P4, F7, F8, T3, T4, T5, T6, O1, O2, Fz, Cz, and Pz, with a fixed reference electrode. The signals were sampled at 1000 Hz and band‐pass filtered at 0.5–70 Hz.

The EEG data were preprocessed in three steps: (i) 60 s of raw EEG data were extracted from each seizure onset time point, resulting in 255 nonoverlapping 60‐s epochs across 23 pediatric patients; (ii) to reduce computational complexity, we averaged 1000 samples in 1 s to represent the signal recording for each second, and this resulted in a matrix with 19 columns (electrodes) and 60 rows (seconds) for each epoch; (iii) to balance each child's data, we employed the bootstrap method to expand the dataset, which resulted in 56 epochs per child and a total of 1288 epochs. Thus far, the discovery cohort comprised 896 epochs, with 560 for responders and 336 for nonresponders, while the testing cohort had 392 epochs, with 280 for responders and 112 for nonresponders. These epochs were used as subjects for subsequent analyses.

### 2.3 Brain network connectivity features extraction

After data preprocessing, we calculated partial correlation measures to estimate functional brain connectivity. A partial correlation is defined as the strength of the relationship between two signals while controlling the effect of one or more other signals; this was previously estimated via three different methods: linear regression, recursive formula, and matrix inversion. Due to the estimation based on regression and recursive approaches being inefficient in terms of computational time and often failing due to the multicollinearity among electrode time series, using the precision matrix, which is also known as the inverse of the covariance matrix, is a more efficient way of estimating the complete set of partial correlations, where off diagonals of a precision matrix have a one‐to‐one correspondence with partial correlations.^26^ In addition, considering the EEG data as time series data and the functional brain network as changing over time, we utilized a novel dynamic statistical model proposed by Zhou et al.^27^ to estimate the partial correlations, which includes three steps. Initially, we calculated the time‐varying data covariance matrix for columns of the data in each epoch, which can be formulated as:

$$\hat{\sum }\left(t\right)=\frac{\sum_{i}\;\omega_{\mathrm{it}}X_{\cdot i}{\left(X_{\cdot i}\right)}^{⊤}}{\sum_{i}\;\omega_{\mathrm{it}}}$$

where $X_{\cdot i}$ is the *i*th column of the data in each epoch and $\omega_{\mathrm{it}}=K\left(|i-t|/\sqrt[{3}]{n}\right)$ is calculated by a Gaussian kernel over time, *t* represents the time point in each epoch from 1 to 60, and *n* is the sample size. Subsequently, we calculated the average covariance matrix for each participant over a single epoch (60 s), and utilized the graphical Lasso method^28^ in the R package “glasso” to estimate the precision matrix per epoch based on the averaged covariance matrix. Finally, based on the definition of partial correlation, we scaled the precision matrix to obtain the complete set of partial correlations for EEG data in each epoch. Thus far, 171 brain network features (edges) had been extracted and were used for the subsequent analyses.

### 2.4 Significant differential network identification

To lower the complexity of our model and make it easier to comprehend, we selected some critical edges from all 171 brain network edges. In this section, we employed the network‐based statistic (NBS)^29^ method to identify differences in brain networks between VNS responders and nonresponders, where the NBS approach is a powerful statistical method that can effectively identify significant differences in networks.

In our study, the NBS analysis was implemented using the R package “NBR,” which consists of three steps. First, based on the data from the two groups, we fitted a general linear model to each edge and computed corresponding *p* values, and suprathreshold edges were initially selected via a user‐specified threshold (*p* < 0.05). Then, the breath‐first‐search algorithm^30^ was used to identify connected components that might be present in the set of suprathreshold edges. Finally, this study used a nonparametric permutation approach to obtain an FWE‐corrected *p* value with 5000 permutations.

After NBS analysis, 90 critical edges were selected for subsequent classification analyses. The selected edges represent significant differences in brain networks between VNS responders and nonresponders, and their analysis provided valuable insights into the underlying mechanisms of the VNS therapy.

### 2.5 Classification model construction and evaluation

The selected 90 key edges were used as input features of a machine‐learning method to classify responders and nonresponders. In this study, we employed the support vector machine (SVM) method,^31^ since this classifier is one of the most robust and accurate classification algorithms, and can efficiently perform nonlinear classification using kernel functions that implicitly map all features into high‐dimensional feature spaces.

In detail, SVM is a supervised machine‐learning method and attempts to use a maximum margin hyperplane to build a generalized linear classifier to distinguish binary data. We trained and validated the SVM model using fivefold cross‐validation in the discovery cohort with the selected 90 edges. Additionally, the optimal tuning parameter combination (“cost” and “gamma”) was determined through the use of a grid search method, which was based on minimizing the average AUC value obtained from the fivefold cross‐validation. By selecting the optimal tuning parameter combination in this way, the model's performance was maximized, thereby ensuring its robustness and reliability in accurately classifying responders and nonresponders. At last, to illustrate the classification ability of SVM at varying discrimination thresholds, we plotted the receiver operating characteristic (ROC) curves. SVM classifiers were constructed in R using the “e1071” package, with the “radial” kernel selected as the kernel function. To account for the minor imbalance between the responder and nonresponder groups, the “class.weight” parameter was specified using the ratio of the two groups.

Moreover, while the SVM method is effective, it may not perform optimally when dealing with large‐scale datasets, consisting of several thousand samples with hundreds of features. To account for the potential challenge of working with such large datasets, we also employed the XGBoost method in our analysis. For a comprehensive overview of our findings and their implications, including those derived from both the discovery and testing cohorts, refer to Section S2 in the Appendix S1.

### 2.6 Testing classification model

In order to assess the reliability and generalizability of our model, we conducted external testing on a separate cohort of participants. Specifically, we applied the trained SVM classifier to the testing cohort consisting of 392 epochs from seven children. Our objective was to use the model to predict the children's responses to VNS therapy and then compare these predicted results to their actual VNS response outcomes. To ensure consistency, we followed the same preprocessing and feature extraction procedures as we had done for the discovery cohort, and inputted the same features into the final classifier. Note that, the parameter combination (“cost” and “gamma”) for the SVM model was chosen in the previous section using cross‐validation and is being applied here.

**FIGURE 1:**
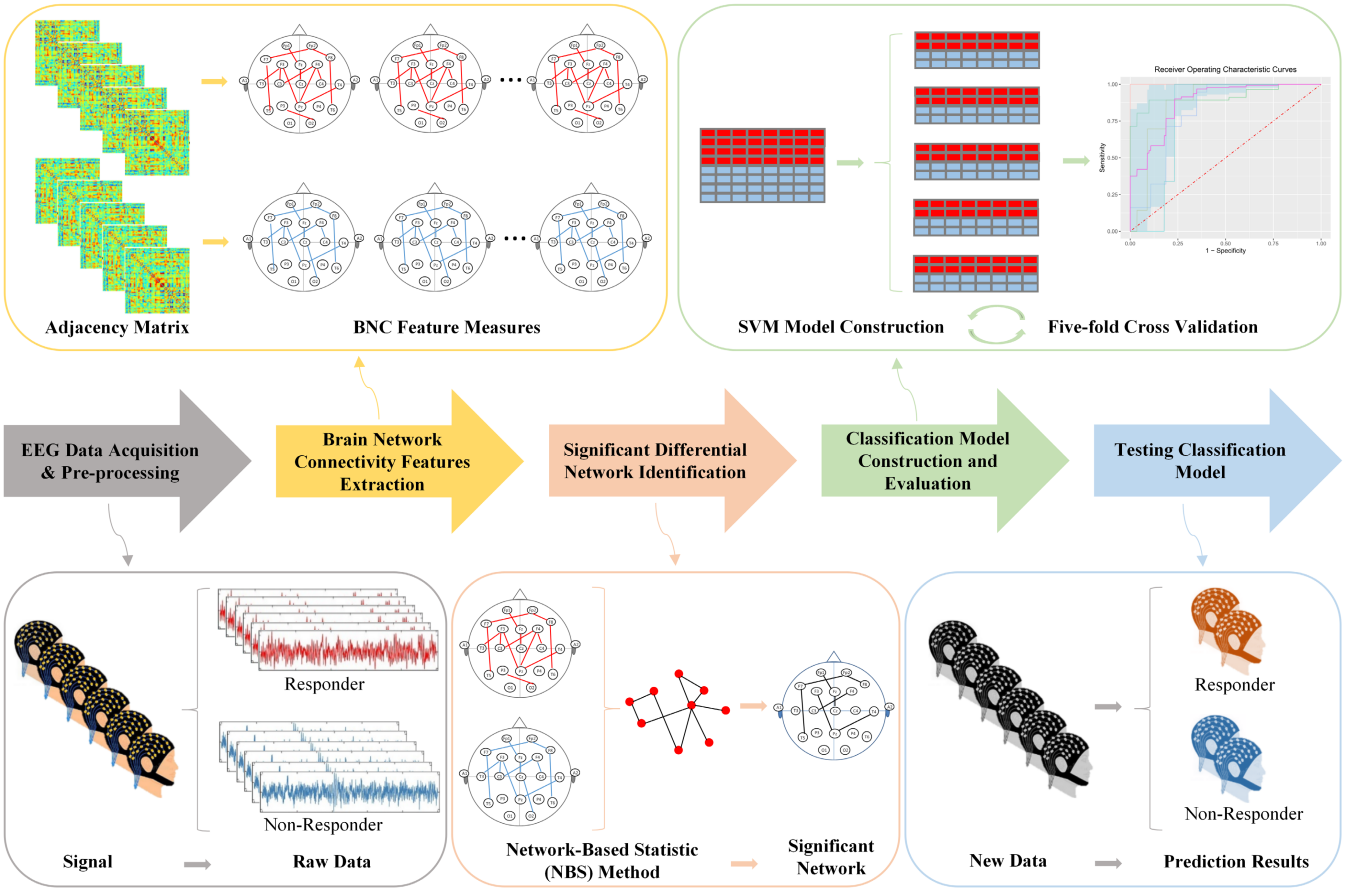
Research methodology flowchart. The flowchart shows all steps of our method in this research.

The methodology flowchart for this research, which outlines the systematic step‐by‐step process of our analysis, is provided in Figure 1. This figure serves as a useful visual representation of our approach, and allows for easy comprehension of the various steps of our study.

## 3 RESULTS

### 3.1 Patient demographics and clinical phenotypes

Twenty‐three pediatric patients were included in our study, including both the discovery cohort and the testing cohort, of which 15 (65%) achieved a positive seizure response to VNS therapy (>50% reduction in main seizure frequency from baseline) and 8 (35%) had a negative or no seizure response to VNS therapy. Table 1 shows subject demographics, seizure characteristics, neuroimaging findings, and outcomes. All continuous variables were analyzed by Kolmogorov–Smirnov test to evaluate data distribution. Patient demographics information and clinical phenotypes were analyzed using the Mann–Whitney test for continuous variables and Fisher's exact test for nominal data.

**TABLE 1:** Overview of pediatric patients' characteristics, stratified by the response to VNS therapy.

Clinical variable	Responder	Nonresponder	*p*
Number of patients (*n*)	15	8	–
Female (*n*, %)	4 (26.7%)	2 (25.0%)	>0.99^a^
Seizure onset age (years, mean ± SD)	1.2 ± 1.6	0.9 ± 0.7	0.92^b^
Epilepsy duration (years, mean ± SD)	3.4 ± 1.9	2.6 ± 1.2	0.56^b^
Follow‐up (years, mean ± SD)	2.8 ± 0.3	2.8 ± 0.3	0.50^b^
Generalized epilepsy (*n*, %)	8 (53.3%)	3 (37.5%)	0.67^a^
Etiology unknown (*n*, %)	8 (53.3%)	3 (37.5%)	0.40^a^
Presence of an MRI lesion (*n*, %)	6 (40.0%)	5 (62.5%)	0.40^a^
Total number of ASMs (*n*, mean ± SD)	7.0 ± 3.5	7.9 ± 3.3	0.56^b^
Pre‐VNS seizure frequency (*t* per day, mean ± SD)	964.5 ± 1368.7	808.6 ± 796.4	0.97^b^
Post‐VNS seizure frequency (*t* per day, mean ± SD)	47.5 ± 83.8	779.2 ± 730.4	0.00025^b^
Seizure reduction rate (%, mean ± SD)	91.2 ± 12.9	−18.7 ± 81.1	0.00011^b^

Abbreviations: *n*, number; SD, standard deviation; *t*, time.

^a^ *p* was calculated using the Fisher exact test.

^b^ *p* was calculated using the Mann–Whitney test.

According to Table 1, there were no significant statistical differences between responders and nonresponders with respect to any demographic, seizure, and imaging covariates, including gender, seizure onset age, epilepsy duration, follow‐up duration, seizure type, causes of epilepsy, presence of an MRI lesion, the total number of ASMs, and pre‐VNS seizure frequency (all *p*s > 0.05). Only outcome covariates (post‐VNS frequency and seizure reduction rate from pre‐ to post‐VNS treatment) exhibited significant differences between the two groups of patients (both *p* < 0.05).

### 3.2 Brain functional connectivity associated with VNS responsiveness

**FIGURE 2:**
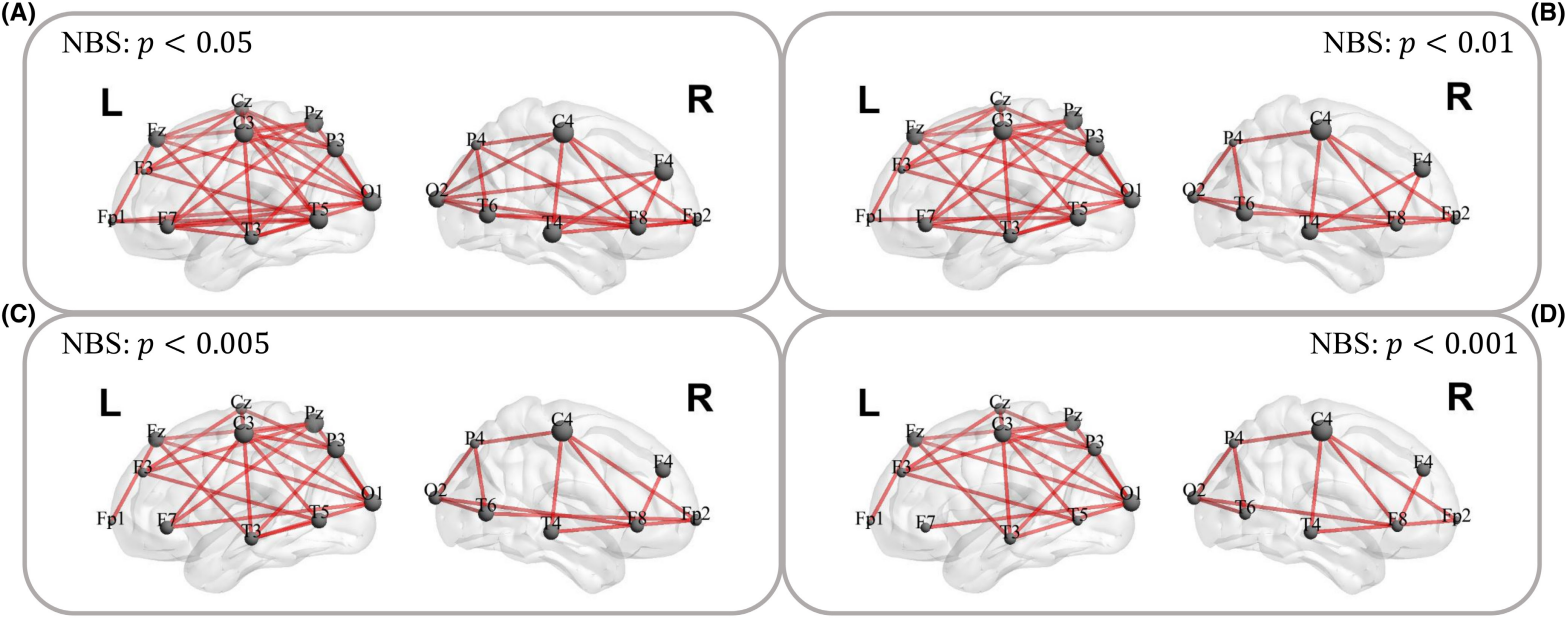
Significant whole‐brain functional connectivity differences between responders and nonresponders with different thresholds in NBS including (A) *p* < 0.05, (B) *p* < 0.01, (C) *p* < 0.005, and (D) *p* < 0.001. L, left; R, right. Gray nodes represent electrode position and red lines represent differential edges between two groups where the larger the gray node, the more differential edges connected to that node.

After the whole‐brain functional connectivity analysis by the dynamic partial correlation estimation procedure, the functional brain network had 171 edges. The NBS method was used to determine the VNS responsiveness‐related functional connectivity based on these edges. In our study, we employed four different thresholds in the NBS method to identify significant differences between responders and nonresponders, in order to ensure the robustness of our results. These four thresholds were selected based on commonly used *p* values in standard statistical analysis practices, namely 0.05 (*p* < 0.05), 0.01 (*p* < 0.01), 0.005 (*p* < 0.005), and 0.001 (*p* < 0.001). The NBS method identified 90, 76, 71, and 62 edges in the differential brain functional network when these thresholds were applied, respectively. Figure 2 shows structures of the differential brain functional network for various NBS thresholds. As can be seen, four differential brain functional networks were roughly identical, suggesting that many overlapping edges were consistently associated with seizure responses to VNS.

Our study identified not only critical edges between two groups of brain functional connectivity but also hub nodes; hub nodes were defined as nodes with high connectivity to other nodes. Table 2 summarizes the top three hub nodes identified by the NBS method under various thresholds, and the most significant connectivity differences between the two groups were identified mainly in the parietal and occipital lobes, primarily related to the postcentral gyrus in the parietal lobe. For the different thresholds used in the NBS method, C4 and C3 were always identified as the top two critical nodes, and the nodes they connected accounted for roughly more than half of the total nodes.

**TABLE 2:** Top three hub nodes identified by the NBS method with four different thresholds. The number of nodes connected to them/total number of nodes is in parentheses.

Rank	NBS: *p* < 0.05	NBS: *p* < 0.01	NBS: *p* < 0.005	NBS: *p* < 0.001
1	C4 (11/19)	C4 (11/19)	C4 (11/19)	C4 (12/19)
2	C3 (9/19)	C3 (10/19)	C3 (10/19)	C3 (11/19)
3	Pz (9/19)	Pz (10/19)	Pz (10/19)	O1 (11/19)

*Note*: Anatomical locations: C4 (R PL postcentral gyrus); C3(L PL postcentral gyrus); Pz (M PL superior‐parietal Lobule); O1 (L OL middle‐occipital Gyrus);

Abbreviations: L, left; M, middle; OL, occipital lobe; PL, parietal lobe; R, right.

### 3.3 SVM classifiers to predict VNS response

As the dataset used in the SVM classifier was based on epochs and each patient had 56 epochs, there were 896 and 392 epochs included in the discovery and testing cohorts, respectively. These epochs were considered as samples in SVM classifiers. Then, AUC, sensitivity, and specificity values were calculated.

**FIGURE 3:**
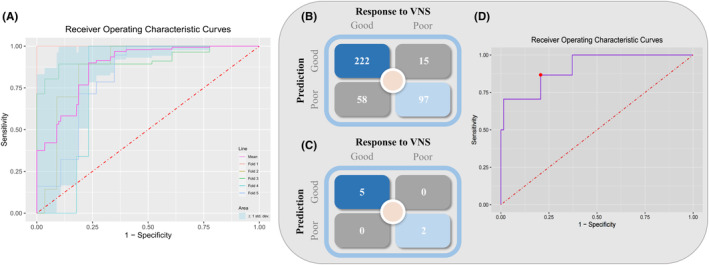
SVM classifiers accurately classify responses to VNS based on brain functional connectivity features. (A) The ROC curve of the SVM classifier for the discovery cohort shows high accuracy on fivefold cross‐validation. (B) Confusion matrix of optimal SVM classifier based on brain functional connectivity features for 392 epochs and (C) for the seven patients in the testing cohort. (D) The ROC curve for identifying VNS responders within the testing cohort (AUC = 0.91) and the red dot represents the current classifier with the optimal cutoff value.

First, for the discovery cohort, fivefold cross‐validation was used to examine the effect of changes in the training data on the SVM classifier output. Figure 3A shows the ROC curves of the SVM classifier in the discovery cohort of different datasets created from fivefold cross‐validation (Fold 1: AUC: 1, sensitivity: 100%, specificity: 100%; Fold 2: AUC: 0.87, sensitivity: 89.3%, specificity: 81.3%; Fold 3: AUC: 0.92, sensitivity: 89.3%, specificity: 90.2%; Fold 4: AUC: 0.79, sensitivity: 100%, specificity: 76.8%; Fold 5: AUC: 0.81, sensitivity: 78.6%, specificity: 73.2%; Mean: AUC: 0.88, sensitivity: 91.4%, specificity: 84.3%).

Then, the SVM classifier was tested using the testing cohort. In this cohort, 280 and 112 epochs were from five responders and two nonresponders, respectively. Our model correctly predicted outcomes of response/nonresponse to VNS therapy for 319 (an accuracy rate of 81.4%) epochs, with an AUC value of 0.91, a sensitivity rate of 86.6%, and a specificity rate of 79.3% (Figure 3B,D). In addition, if more than half (28) of the epochs for a patient were classified as responder or nonresponder, our model classified this patient as responder or nonresponder, respectively. Finally, at the patient level, our model correctly predicted all patients in the testing cohort and achieved 100% accuracy (Figure 3C).

## 4 DISCUSSION

In recent years, VNS has been proven to be an effective and safe therapy for pharmacoresistant epilepsy in children. There is accumulating evidence suggesting that VNS therapy can reduce seizure frequency. However, it is still impossible to accurately predict children who will benefit from VNS or fully elucidate the mechanistic roles that explain the potential role of VNS in the treatment of epilepsy. Consequently, there is an urgent need to investigate suitable novel methods to select target pediatric patients and explore the mechanisms of action of VNS. Here, we explore the relationship of brain functional connectivity using preoperative scalp EEG with seizure response to VNS and provide the first brain functional connectivity based analysis procedure using scalp EEG data to predict the responsiveness to VNS. Based on the intrinsic characteristics of EEG, seizure epochs were picked up from the overall scalp EEG data of each patient as samples for our study. Then we used partial correlation values between every two electrodes to measure brain functional connectivity.

This study presents several novel findings, including (i) we propose a novel method to extract brain functional connectivity features using scalp EEG data during seizures and found significant differences in functional brain connectivity between VNS responders and nonresponders, (ii) the differential analysis of functional brain connectivity between responders and nonresponders reveals that the connections of the postcentral gyrus located in the right and left parietal lobe regions are most associated with VNS response, (iii) we constructed a predictive model based on SVM learning that can perform well on patient‐level cross‐validation and accurately predict VNS responders on an independent testing cohort under both epoch level and patient level. These findings can expand the understanding of the mechanism of action of VNS, provide support for determining the target children for VNS therapy, and have important implications for the treatment of intractable epilepsy in children.

### 4.1 Brain functional connectivity affecting VNS response

In recent years, several theories have been proposed to explain the mechanism of action of VNS from the brain functional network perspective. Based on fMRI data, Ibrahim et al.^18^ conducted a whole‐brain connectivity analysis using correlation coefficients between the time series of thalamic regions and the time series of all brain voxels in functional space and found stronger thalamocortical connectivity in patients with good response to VNS. Based on MEG data, Babajani‐Feremi et al.^17^ used phase locking value (PLV) to measure the brain functional connectivity and then utilized this to calculate three global brain network measures; they reported that the brain functional network topologies were significantly different in VNS responders compared to nonresponders. Mithani et al.^16^ investigated the band‐limited brain functional connectivity using the weighted phase lag index (wPLI) and found that changes in functional brain connectivity were associated with the VNS response. Based on EEG data, Fraschini et al.^21^ used the phase lag index (PLI) to construct a brain network and presented a reorganization of the brain network in VNS responders. More recently, Sangare et al.^32^ employed both PLI and PLV to estimate the brain networks and confirmed that VNS could modulate functional connectivity. The results of these studies provide some evidence that brain functional connectivity may affect VNS response.

In the current study, we first used ictal EEG recording to construct a brain functional network, and our results revealed significant differences in functional brain connectivity between VNS responders and nonresponders, which were consistent with the findings of previous studies. To fully demonstrate the existence of these differences, we also tried different thresholds in the NBS method and selected at least 62 edges as significantly different out of the 171 edges. These distinct connections in the brain network are mainly related to electrodes C4 and C3.

### 4.2 Hub brain regions associated with VNS response

Electrodes C4 and C3 corresponded to the postcentral gyrus located in the left and right parietal lobe regions according to anatomical locations of the international 10–20 cortical projection points proposed by Okamoto et al.^33^ According to the definition of brain hubs,^34^ they can be considered hub brain regions associated with VNS response.

Thus far, a series of recent studies on epileptic brain network hubs have shown that hub identification can provide insight into network alterations in epilepsy, help to understand the mechanisms of seizure spread, and provide markers for postoperative seizure recurrence in drug‐resistant patients.^35^, ^36^, ^37^, ^38^, ^39^, ^40^ However, no studies have used ictal data to identify brain network hubs associated with postoperative outcomes. To our knowledge, the current study is the first to identify brain functional network hubs related to VNS response.

As the actual mechanism of action of VNS in pediatric patients is unknown, no gold standard is available to evaluate the hub identification results from our study. We found experimental support for brain functional network hubs associated with VNS response. For example, the postcentral gyrus is a prominent gyrus in the lateral parietal lobe of the human brain, the location of the primary somatosensory cortex, and the main sensory receptive area for touch. By reviewing previous mass studies, Royer et al. discovered that the lateral parietal lobe is a high‐centrality region in the brain and pointed out that impaired connectivity in this region could contribute to epilepsy.^35^ Furthermore, Narayanan et al.^41^ detected VNS‐induced activation in the postcentral gyrus, which suggested that this brain area may play a role in modulating cerebral cortical activity, and the observed decrease in seizure frequency in VNS responders may be a consequence of this changed activation. Recently, Zhu et al. also confirmed that VNS response was associated with significant brain network alternation in the postcentral gyrus.^42^, ^43^ These results confirm our findings supporting the role of connections related to the postcentral gyrus in VNS efficacy.

### 4.3 Prediction of pediatric patient response to VNS based on brain functional connectivity

Although VNS implantation is a relatively safe procedure, it still carries certain risks, especially in nonresponders. The most common complications include postoperative hematoma, infection, and vocal cord palsy. Therefore, accurate identification of VNS responders is a critical unmet need as it can both prevent undue harm and save medical resources and reduce disappointment for VNS nonresponders.

In the current study, we first used ictal scalp EEG recordings to estimate functional brain connectivity measures and utilized them as input to construct a predictive model based on SVM learning. To ensure the reliability of our model, we first examined the performance in the discovery cohort via fivefold cross‐validation and then validated this in an independent testing cohort. Our model attained >80% accuracy under the epoch level in the testing cohort and achieved 100% under the patient level. We also compared the prediction performance of our study with those of previous studies (Mithani et al.: accuracy = 89.5% at patient level^16^; Babajani‐Feremi et al.: accuracy = 87% at patient level^16^; Ibrahim et al.: accuracy = 86% in patient level^18^); our model outperforms them all.

Our study stands out for three novelties. First, previous studies mainly used fMRI and MEG data. However, to our knowledge, this is the first study to use EEG data, which is more common in the epilepsy field, to predict VNS responders. Second, this is also the first study to use ictal EEG recordings to identify potential VNS responders, whereas other studies used resting state data. Third, we proposed a dynamic partial correlation method to capture the functional brain connectivity, which can prevent spurious correlations and engender more reliable results in prediction.

### 4.4 Limitations and future directions

The current research has the following three limitations. First, the sample size of patients is relatively small (*n* = 23), which may lead to unreliable prediction results. Nevertheless, this is unlikely to have affected our study's results for several reasons: (i) our predictive model was constructed under the epoch level, and for each patient, there were 56 epochs extracted from raw EEG data directly or generated by data augmentation, which is enough for prediction with fewer than 100 predictors; (ii) to avoid overfitting due to limited dataset size, we performed a fivefold cross‐validation analysis, one of the most widely used resampling techniques, to test the model's ability to predict new patients' data that were not used for estimation.

However, we acknowledge that the performance of our prediction model should also be evaluated in the future with new data utterly independent of the training dataset. In details, in the future, we plan to increase the sample size of our dataset to enhance the statistical power of our analysis and minimize the risk of bias. Moreover, we also intend to incorporate more diverse data sources and utilize advanced modeling techniques to extract more intricate patterns and enhance the overall accuracy and generalizability of our predictions.

Another limitation is that the SVM classifier in the current study assumes that all brain functional connectivity features of the discovery cohort contribute equally to predicting VNS response. It is expected that in real‐world situations, some of these features may be more correlated, while other features may be less correlation with VNS response. However, we presume that the weights of these features are minimized in our study since (i) the number of significant features obtained by the NBS method with different thresholds was similar, indicating that the weight difference of input features was small and would not affect the prediction results; and (ii) the predictive performance of the current study was satisfactory, and our model outperformed most existing studies.

Nevertheless, we have identified two future directions to address this limitation: (i) we plan to propose a new feature‐weighted machine‐learning classifier to improve the accuracy of our future results; (ii) we expect to perform a more detailed analysis of the functional connectivity features to identify which features are most strongly associated with VNS response, and then incorporate this information into the classifier.

Another limitation of this study is that our entire cohort had more responders than nonresponders. Although we used the “class.weight” parameter in implementing the SVM method and most of existing studies have such restrictions, future prospective balanced cohorts are desirable to validate our findings to identify children who may benefit from VNS.

## 5 CONCLUSIONS

Vagus nerve stimulation – as a low‐risk surgical treatment – is used for pediatric patients with pharmacoresistant epilepsy. Accurately predicting patients who will respond to VNS therapy has been a challenge to date. There is a persistent need to identify potential responders to VNS preoperatively. This study generated a classifier using functional brain connectivity features extracted from seizure duration in EEG data to distinguish VNS responders from nonresponders accurately. In addition, our findings reveal that more connections involved in the postcentral gyrus of the left and right parietal lobe regions might contribute to knowledge regarding the mechanisms of action of VNS. In conclusion, we demonstrate that brain functional connectomics may contain important information related to VNS response. We look forward to being validated in larger cohorts to reduce unnecessary harm to children, help clinicians select optimal treatment strategies, and improve patients' prognoses.

## AUTHOR CONTRIBUTIONS

Hao Chen and Yi Wang contributed to the study methodology, software, formal analysis, visualization, and writing – original draft. Taoyun Ji contributed to the study conceptualization, formal analysis, and writing – review and editing. Yuwu Jiang and Xiao‐Hua Zhou supervised, carried out project administration, writing – review and editing, and acquired funding.

## FUNDING INFORMATION

This work was supported by the National Key R&D Program of China (2021YFF0901400), the National Natural Science Foundation of China (12026606, 12171279), the China Postdoctoral Science Foundation (2021M700249), the National High Level Hospital Clinical Research Funding (Multi‐center Clinical Research Project of Peking University First Hospital) (2022CR60), and Novo Nordisk A/S.

## CONFLICT OF INTEREST STATEMENT

The authors declare that they have no conflict of interest.

## CONSENT TO PARTICIPATE

The parents of the patients signed written informed consent and agreed with their children's participation in this study and allowed the use of the relevant data and information for scientific research.

## Supporting information


Data S1

## Data Availability

The datasets generated and analyzed during the current study are available from the corresponding author upon reasonable request.
