# Supplementary material for: Brain functional connectivity‐based prediction of vagus nerve stimulation efficacy in pediatric pharmacoresistant epilepsy
Source: CNS Neurosci Ther. 2023 May 11;29(11):3259–68. doi: 10.1111/cns.14257 (PMC10580342; doi:10.1111/cns.14257)
Supplement: Supplementary file 1 — Data S1 [file CNS-29-3259-s001.docx]

**Supplementary Material for “Brain Functional Connectivity-based Prediction of Vagus** **Nerve Stimulation Efficacy in Pediatric Pharmaco-resistant**

**Epilepsy”**

Hao Chen1#, Yi Wang1#, Taoyun Ji2#, Yuwu Jiang2*, Xiao-Hua Zhou134*

1 Beijing International Center for Mathematical Research, Peking University, Beijing, China

2 Department of Pediatrics and Pediatric Epilepsy Center, Peking University First Hospital, Beijing, China

3 Department of Biostatistics, School of Public Health, Peking University, Beijing, China

4 Pazhou Lab, Guangzhou, China

* Correspondence: Xiao-Hua Zhou, Yuwu Jiang <azhou@math.pku.edu.cn, jiangyuwu@bjmu.edu.cn>

# These authors have contributed equally to this work and share ﬁrst authorship

**S1. Details of the EEG data acquisition and pre-processing**

The Scalp EEG data used herein were collected before VNS implantation. Its signals were recorded using a 19-channel EEG system positioned according to the 10-20 system, including FP1, FP2, F3, F4, C3, C4, P3, P4, F7, F8, T3, T4, T5, T6, O1, O2, Fz, Cz, and Pz, with a ﬁxed reference electrode, and the signal recording for each electrode is obtained as potential electrical diﬀerences between itself and the reference electrode. Acquired signals were digitized at a sampling rate of 1000 Hz and successively band-pass ﬁltered at 0.5–70 Hz.

The pre-processing procedure of EEG data was divided into the following three steps. First, we extracted 60 seconds of raw Scalp EEG data from each seizure onset time point for each subject, annotated by certiﬁed epileptologists (T.J.), generating 255 non-overlapping 60-second epochs across 23 pediatric patients for analysis. Then, to reduce computational complexity, we averaged 1,000 samples in one second to represent the signal recording for one second. Thus far, the data was in matrix form with 19 columns (electrodes) and 60 rows (seconds) for each epoch. Subsequently, due to the seizure frequency of each child recorded by Scalp EEG varying from 2 to 56, we used the bootstrap method to conduct data augmentation to keep the data balanced. For each participant, we performed sampling with replacement to create a new dataset of 56 epochs. Thus far, there were 1,288 epochs, including 896 epochs in the discovery cohort (560 for responders and 336 for non-responders) and 392 epochs in the testing cohort (280 for responders and 112 for non-responders), and employed epochs as subjects in subsequent analyses.

**S2. The results of XGBoost classifiers for VNS response prediction**

In order to address the possibility of encountering large sample situations, we utilized the XGBoost method [1] for our dataset, which consisted of 896 and 392 epochs in the discovery and testing cohorts, respectively. Results from both the discovery and testing cohorts are presented below.

Within the discovery cohort, we conducted a five-fold cross-validation to assess the impact of changes in the training data on the SVM classifier output and to determine the optimal tuning parameters for use in the testing cohort. The outcomes for the different folds of cross-validation are as follows: Fold 1 had an AUC of 0.91, a sensitivity of 94.6%, and a specificity of 81.3%; Fold 2 had an AUC of 0.86, a sensitivity of 78.6%, and a specificity of 94.6%; Fold 3 had an AUC of 0.90, a sensitivity of 100%, and a specificity of 80.4%; Fold 4 had an AUC of 0.82, a sensitivity of 83.9%, and a specificity of 66.1%; Fold 5 had an AUC of 0.78, a sensitivity of 78.6%, and a specificity of 71.4%. The mean values were: AUC of 0.85, sensitivity of 87.9%, and specificity of 79.7%. Subsequently, using the optimal tuning parameters identified, the results from the testing cohorts were as follows: an accuracy rate of 79.1%, an AUC value of 0.90, a sensitivity of 92.0%, and a specificity rate of 73.9%.

By comparing the results obtained by the SVM model in our paper, we found that these two methods are both employed effectively in our study. Therefore, we believe that if there is a large-scale dataset, readers can use XGBoost as a reliable replacement method.

**S3. Discussion of the utility of partial correlation measures in constructing brain functional connectivity through scalp EEG data**

Following the reviewer's recommendation, we will discuss the reasoning behind using partial correlation measures to construct brain functional connectivity through scalp EEG data in this section. The brain is a complex network comprising interconnected regions, and comprehending the functional connectivity among these regions is crucial to understanding brain function. Scalp EEG is a non-invasive technique for measuring brain activity that has been extensively employed to investigate brain functional connectivity.

Traditionally, many researchers in the field of EEG data analysis have used synchronization metrics to quantify brain functional connectivity. These metrics measure the degree of synchronization or coherence between pairs of EEG electrical nodes. For example, the magnitude squared coherence (MSC) measures the degree of linear relationship between two EEG electrical nodes [2], while the phase locking value (PLV) measures the degree of phase synchronization between two EEG electrical nodes [3].

However, in recent years, there has been increasing interest in using partial correlation measures to construct brain functional connectivity networks from EEG data [4,5,6,7]. The main reason for this shift is that traditional synchronization metrics are confounded by the influences of other sources of EEG activity, such as common sources of noise or reference electrode signals. By contrast, partial correlation measures are used to estimate the relationship between two EEG signals after removing the effects of other signals, which can help to isolate the specific functional connections between different brain regions and construct more precise and accurate functional connectivity networks from EEG data.

In addition, volume conduction can be viewed as a significant confounding factor in the interpretation of results in the construction of functional connectivity networks from EEG data, where many traditional synchronization metrics, such as coherence and phase-locking value, are sensitive to volume conduction effects and can result in false positive connections in the functional connectivity network. Partial correlation analysis is a method that can handle volume conduction effects by removing the effects of other sources of EEG activity, such as common sources of noise or reference electrode signals. By estimating the partial correlation between pairs of EEG signals, researchers can construct a brain functional connectivity network that represents the specific functional connections between different brain regions, independent of other sources of EEG activity. Recently, there was an existing literature proposed by Dai [8] support this opinion that partial correlation may help to eliminate the effect of volume conduction on EEG data and thus obtain an accurate functional connectivity network, which proposed that using partial correlation to build the functional connectivity network would provide an excellent performance against volume conduction in both simulated and human EEG.

In conclusion, partial correlation measures are a valuable tool for constructing brain functional connectivity networks from EEG data. They enable researchers to obtain more accurate estimates of the true connectivity between brain regions, construct directed functional connectivity networks, and remove the impact of volume conduction.

**S4. Additional experiments using different epoch length**

As per the reviewer's suggestion, we conducted additional experiments in this section using various epoch lengths to demonstrate that the epoch length used in our paper (60-second) is optimal. Firstly, we would like to explain our selection criteria for the epoch length. Our EEG data analysis revealed that seizures typically lasted 15 to 60 seconds. Therefore, we aimed to include more seizure information to obtain a more accurate estimation of the partial correlation matrix. Consequently, we defined 60 seconds as the epoch length for our study.

We acknowledge that shorter epoch lengths offer higher temporal resolution and can capture more details about changes in brain activity over time, while longer epoch lengths can reduce noise by averaging out variability across a larger time window. In our study, we estimated covariance matrices per second using a time-varying method and then averaged 60 of them to obtain the partial correlation matrix for one epoch. Hence, we believe that our method can exploit both shorter and longer epoch lengths.

Next, we experimented with other epoch lengths, i.e., 15s, 30s, and 45s, and report the AUC values for each fold in a 5-fold cross-validation and testing cohort in the Table S1:

| **Table S1.** AUC values for each fold in a 5-fold cross-validation and a testing cohort using different epoch lengths | | | | | | |
| --- | --- | --- | --- | --- | --- | --- |
|  | Fold 1 | Fold 2 | Fold 3 | Fold 4 | Fold 5 | Test |
| 15 s | 0.93 | 0.82 | 0.73 | 0.61 | 0.78 | 0.76 |
| 30 s | 0.92 | 0.81 | 0.73 | 0.78 | 0.81 | 0.79 |
| 45 s | 1 | 0.82 | 0.88 | 0.71 | 0.86 | 0.87 |
| 60 s | 1 | 0.87 | 0.92 | 0.79 | 0.81 | 0.91 |

We observed that as the epoch length increased, the variability of AUC values in cross-validation decreased while the AUC values for the testing cohort increased. These trends are both logical and desirable. Based on these findings and facts, we conclude that an epoch length of 60 seconds is ideal for our study.

# References

[1] Chen T, Guestrin C. Xgboost: A scalable tree boosting system. *Proceedings of the 22nd acm sigkdd international conference on knowledge discovery and data mining*. 2016;785-794. doi:10.1145/2939672.2939785

[2] Shan X, Cao J, Huo S, et al. Spatial–temporal graph convolutional network for Alzheimer classification based on brain functional connectivity imaging of electroencephalogram[J]. *Hum Brain Mapp*. 2022;43(17):5194-5209. doi:10.1002/hb m.25994

[3] Darvas F, Ojemann JG, Sorensen LB. Bi-phase locking—a tool for probing non-linear interaction in the human brain. *Neuroimage*. 2009;46(1):123-132. doi:10.1016/j. neuroimage.2009.01.034

[4] Akın A. Partial correlation-based functional connectivity analysis for functional near-infrared spectroscopy signals. *J Biomed Opt*. 2017;22(12):126003-126003. doi: 10.1117/1.JBO.22.12.126003

[5] Ye Y, Xia Y, Li L. Paired test of matrix graphs and brain connectivity analysis. *Biostatistics*. 2021;22(2):402-420. doi:10.1093/biostatistics/kxz037

[6] Xia Y, Li L. Hypothesis testing of matrix graph model with application to brain connectivity analysis. *Biometrics*. 2017;73(3):780-791. doi:10.1111/biom.12633

[7] Hlinka J, Alexakis C, Diukova A, et al. Slow EEG pattern predicts reduced intrinsic functional connectivity in the default mode network: an inter-subject analysis. *Neuroimage*. 2010;53(1):239-246. doi:10.1016/j.neuroimage.2010.06.002

[8] Dai X. Partial correlation and partial cross-correlation as multivariate measures of EEG connectivity. *University of California, Irvine*. 2021.
